# Resting Heart Rate and Auditory Evoked Potential

**DOI:** 10.1155/2015/847506

**Published:** 2015-10-04

**Authors:** Simone Fiuza Regaçone, Daiane Damaris Baptista de Lima, Vitor Engrácia Valenti, Ana Cláudia Figueiredo Frizzo

**Affiliations:** Department of Speech Therapy and Audiology, Faculty of Philosophy and Sciences, UNESP, Avenida Hygino Muzzi Filho 737, 17525-900 Marília, SP, Brazil

## Abstract

The objective of this study was to evaluate the association between rest heart rate (HR) and the components of the auditory evoked-related potentials (ERPs) at rest in women. We investigated 21 healthy female university students between 18 and 24 years old. We performed complete audiological evaluation and measurement of heart rate for 10 minutes at rest (heart rate monitor Polar RS800CX) and performed ERPs analysis (discrepancy in frequency and duration). There was a moderate negative correlation of the N1 and P3a with rest HR and a strong positive correlation of the P2 and N2 components with rest HR. Larger components of the ERP are associated with higher rest HR.

## 1. Introduction

Cardiovascular diseases are the most frequent causes of morbidity and mortality around the world during the last decades [1–4]. The cardiovascular system is influenced by extrinsic stimuli through the autonomic nervous system (ANS) [5]. The central nervous system (CNS) [6] sends commands to the ANS, controlling the regulation of heart rate (HR) and blood pressure (BP); the sympathetic system is responsible for the increase in HR whereas the parasympathetic system is responsible for the decrease in HR.

Since the CNS actively regulates the cardiac system, auditory evoked-related potentials (ERPs) are an important method used to evaluate it because it captures electrical responses in the cortex due to an acoustic stimulus. Brainstem auditory ERPs are characterized by bioelectric responses of thalamic and cortical activity, measured in milliseconds; it assesses cortical activities involved in the skills of discrimination, integration, memory, and attention in the brain, besides the integrity of central auditory nervous system [7].

Few studies have focused on the cardiac and auditory systems through musical stimuli [8–10]; cardiac and auditory potentials [11, 12]; and white noise [13, 14], indicating that there is an association between hearing bioelectrical response and HR regulation. According to the literature, more studies are necessary to confirm these findings, particularly the interaction between auditory evoked potential and cardiac autonomic regulation [15].

Knowledge of physiological responses involved in the relationship between the central auditory pathways and cardiac autonomic regulation is important for the development of future therapies to propose intervention and prevent the development of disorders of the cardiovascular system. In this sense, it is important to have new studies to help to understand the association between cardiac autonomic control and the electrical activity in the auditory system. Therefore, we aimed to evaluate the association between HR and ERPs at rest in women.

## 2. Methods

### 2.1. Subjects

This research protocol was approved by the Ethics Committee in Research of the State University of São Paulo (case number 419/2012) and was in accordance with Resolution 196/96 of the National Health Council of 10/10/1996. The sample consisted of 21 apparent healthy female subjects between 18 and 24 years old. All volunteers were informed about the procedures and objectives of the study and, after agreeing, signed a consent form.

### 2.2. Procedure

We excluded smokers, people with any degree of hearing loss or middle ear disorders, subjects with related cardiorespiratory, metabolic, neurological, and/or any condition that prevented the individual to perform the procedures, and those users of drugs that alter cardiac autonomic regulation. For this characterization, the following procedures were performed: medical history and research of hearing threshold (air and bone conduction) and acoustic impedance measurements; resting blood pressure and heart rate measurement.

The normal parameters of hearing assessments considered in this study were pure tone audiometry thresholds below or equal to 25 dB and airway below or equal to 15 dB bone conduction [16], with acoustic impedance tympanogram type A, indicating eardrum-ossicular normal [17] system and presence of ipsilateral and contralateral reflexes.

Data collection was conducted in a quiet room electric and sound-proof in temperatures between 21°C and 25°C and humidity between 50 and 60%. Volunteers were instructed to not ingest alcohol and caffeine in the 24 hours prior to evaluation. After the initial assessment, the heart rate receiver Polar RS800CX (Polar Electro, Finland) was placed on the chest of the volunteers in the sternum region for analysis. After placing the strap and the monitor, subjects were positioned in a chair and told to remain at rest for 10 minutes. Blood pressure was measured using a sphygmomanometer and stethoscope. Blood pressure and heart rate were measured at rest prior to the auditory ERPs. Only volunteers who showed no significant differences in the values of these two measures participated in the survey.

The examination of the auditory ERPs was performed with the individual in a state of alert watching a video (without sound) for distraction and recommended to not direct their attention to sound stimuli in oddball paradigm (two auditory stimuli, deviant-standard, presented in random order), unattended test. We used Biologic's Evoked Potential System (PE) for data acquisition and five disposable electrodes placed on Fz and Cz in reference to the right lobe (A2) and left (A1) and ground in Fpz, using the two channels of the equipment record.

The parameters used for the ERPs were filter between 0.5 and 30 Hz binaural stimuli in the frequency discrimination, the standard stimulus which was elicited at a frequency of 750 Hz and 1000 Hz for the deviant stimulus (tone burst with plateau 60 ms and rise/fall 20 ms), the duration discrimination on, the standard stimulus (tone burst with plateau 60 ms and rise/fall 20 ms), deviant stimulus (burst tone with plateau 30 ms and rise/fall 10 ms) both in the frequency of 1000 Hz (with a probability of 20%), intervals between stimuli of 1.1 ms, intensity of 70 dB HL, analysis time of 500 ms, prestimulus analyses time of 0 ms, a sensitivity of 100 microvolts alternating polarity, and number samples of 200 stimuli [18].

The wave identification ERPs followed criteria in the literature, including visualization of sequential peaks of negative-positive-negative waves, that is, N1, P2, N2 complex, respectively, between 60 and 300 ms, observed in do twice traces [19]. As the component P3a latency was marked before 350 ms/P3b, in unattended test [20], the P3b component was not analyzed in this study. The P3a is the AEP components related to early warning processes and auditory sensory processing, which occurs automatically in response to the large differences of the stimuli, independent of the active attention of the individual to the stimulus sequence [21].

Testing took approximately 50 minutes. In order to maintain a standard quality test, in the volunteers who showed myogenic interference, we suggested change positions and when necessary the examination was repeated.

The Shapiro-Wilk Test was applied to evaluate distributions; Person and Spearman correlation Tests were applied for parametric and nonparametric distribution, respectively, in order to investigate correlation between variables. The *P* values were considered statistically significant when *P* < 0.05 and all confidence intervals were constructed with 95% statistical confidence. For correlation values we considered strong correlation for *r* > 0.5, moderate correlation for *r* between 0.3 and 0.49 (0.3 > *r* > 0.49), and weak correlation for *r* < 0.3. The statistical software used was GraphPad Software StatMate 2:00 version for Windows, GraphPad Software, San Diego, California USA.

## 3. Results

**Figure 1:**
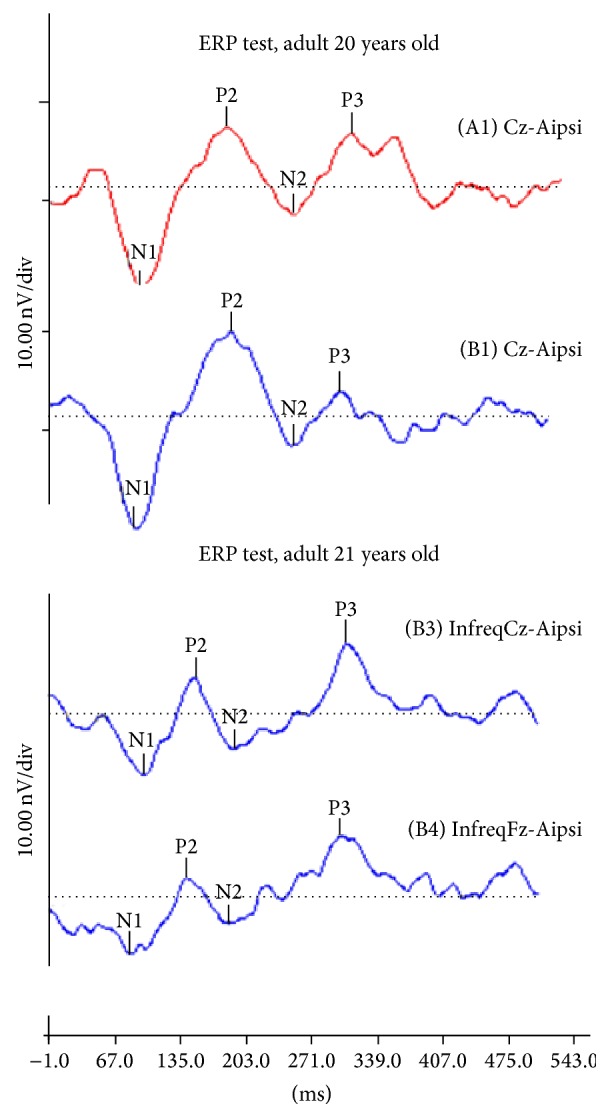
ERP test of two individual adults.

Correlations between latencies and amplitudes of N1, P2, N2, and P3a and N2-P3a interamplitude, measured at Cz and Fz (Figure 1), related to ERP and HR in healthy women were studied.

**Figure 2:**
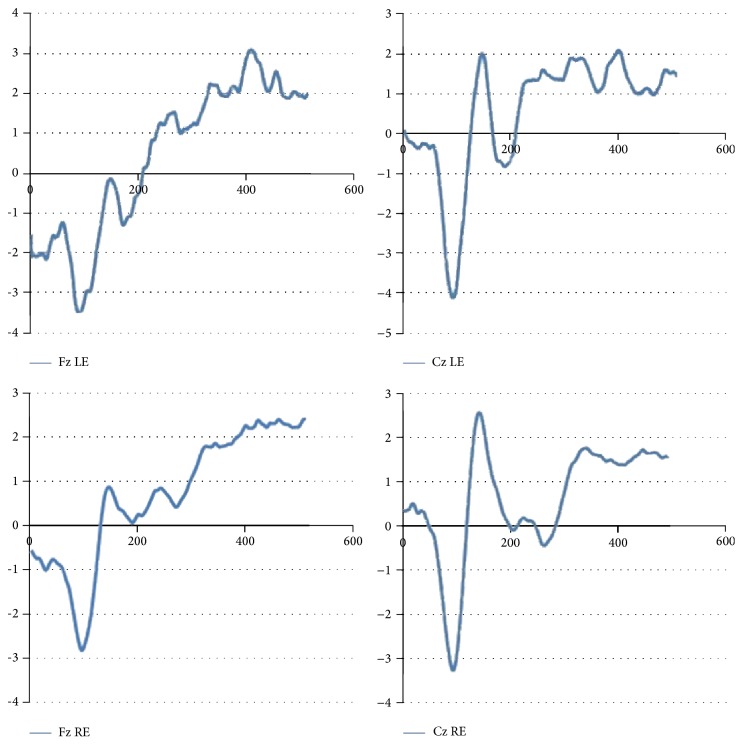
Grand average of ERP tests—frequency protocol. RE: right ear; LE: left ear.

The results obtained show correlation between some components of the ERP and HR as shown in Tables 1, 2, 3, 4 and in Figure 2.

## 4. Discussion

The ERP is one of the promising measures used in the research of central auditory processing that reflects cortical activity involving listening skills from the simple to the most complex. HR is mediated by the direct activity of the ANS through sympathetic and parasympathetic branches and is an indicator usually expressed as the number of heartbeats per minute (bpm).

The findings of the literature state that the decrease or increase in HR is caused by the selective activation of cardiac neurons in the amygdala and in the reticular formation, structures located in the cerebral cortex. The reticular formation is responsible for the regulation of alertness and subsidizes the attentional process and the amygdala is specifically related to emotions [22].

Anatomically and physiologically the link between cardiovascular and hearing impairment due to structures responsible for cardiovascular regulation and components of ERPs that presents similar functions in the regulation of alertness and sensory attentional process is evident. The N1 component of the ERP is associated with attention and initial decoding of the auditory stimulus. The P2 is related to temporal acoustic stimulus, which brings information to the level of the auditory cortex, the early cortical processing of sound and features. The N2 contributes to physical breakdown of the acoustic characteristics of the stimuli and is also responsible for passive, preattentional, automatic perception, discrimination, and sound recognition [7, 18] response activities. The P3a is related to the activity of alert during the initial allocation of attention or redirection of sensory attention and is triggered by distractor stimuli [23, 24].

It was possible to observe in the Cz scan that when the right and the left ear were aurally stimulated, there was a moderate negative correlation in the amplitude of N1 and P3a, respectively. It was reported that when the electrical activity reaches the cortex in areas associated with attention and initial decoding of the stimulus and during stimulation the stimulus starts to be processed in the auditory pathway through activity updates for automatic allocation of sensory attention, while HR increases the amplitude of N1 and P3a decreases.

Some authors [11, 12] related cardiac evoked potentials with long-latency auditory evoked potential and showed that there is a link between CNS and ANS in the reflection of some aspects of processing auditory stimulus, confirming our findings in relation to the auditory N1 component.

Moreover, we observed strong positive correlation of the amplitude of P2 and N2 amplitude and latency with HR in the scan Cz and Fz in the right and left ear. Therefore, the results showed that the lower the electrical activity of the auditory processing is, the lower the HR. P2 and N2 components reflect the acoustic characteristics of the stimuli [18], which can affect the ANS and interfere with the heartbeat.

A study of simulated driving mental fatigue testing associated with another type of auditory cortical potential observed significant reduction in P300 amplitude, indicating that when the individual was exposed to the mental fatigue there was a decrease of attention to the auditory task. The authors suggested that auditory electrophysiological response is related to mental fatigue, which has impact on the function of the CNS, which consequently controls and regulates the cardiovascular system [14].

Finally, regarding the parameters of frequency and duration, we observed statistical correlation only in the frequency protocol in both ears. This fact can be explained because their stimuli were presented in an oddball paradigm and differentiate in intensity, which can make them more discriminating and hence may affect the ANS and interfere with the heartbeat.

## 5. Conclusion

There was association between cardiovascular parameters and the central auditory pathways, indicating physiological responses of the studied variables and the relationship between them. We suggest further research in this field, in order to clarify and confirm this association.

## Figures and Tables

**Table 1:** Correlation between latency (ms) and amplitude (*μ*V), ERPs and HR in frequency protocol on the right ear.

RE	Variables	Mean	SD	*r*	*P*
Cz	LAT N1	98,29	9,72	0,100	0,40
	AMP N1	−4,80	1,80	−0,492	0,02^*^
	LAT P2	169,93	29,84	0,300	0,60
	AMP P2	2,24	1,99	0,200	0,50
	LAT N2	221,63	37,84	0,200	0,50
	AMP N2	−1,77	1,98	0,100	0,70
	LAT P3a	289,05	32,49	0,100	0,40
	AMP P3a	2,03	1,82	0,300	0,10
	AMP N2-P3a	−3,68	3,47	0,090	0,90

Fz	LAT N1	97,85	9,60	0,040	0,30
	AMP N1	−4,45	2,19	0,100	0,30
	LAT P2	157,48	25,18	0,100	0,30
	AMP P2	0,76	2,17	0,557	0,00^*^
	LAT N2	201,04	32,15	0,200	0,50
	AMP N2	−1,49	2,28	0,563	0,00^*^
	LAT P3a	288,16	30,84	0,200	0,50
	AMP P3a	2,06	1,41	0,100	0,40
	AMP N2-P3a	−3,74	3,11	0,090	0,40

RE: right ear; LAT: latency; AMP: amplitude; SD: standard deviation; ^*^ *P* < 0.05; Shapiro-Wilk Test, Person Test, and Spearman Test.

**Table 2:** Correlation between latency (ms) and amplitude (*μ*V), ERPs and HR in duration protocol on the right ear.

RE	Variables	Mean	SD	*r*	*P*
Cz	LAT N1	102,04	12,87	0,010	0,70
	AMP N1	−4,08	1,87	0,100	0,90
	LAT P2	148,41	15,52	0,200	0,20
	AMP P2	1,70	1,75	0,300	0,10
	LAT N2	209,63	20,11	0,070	0,50
	AMP N2	−3,06	1,15	0,200	0,40
	LAT P3a	301,99	23,87	0,100	0,40
	AMP P3a	2,25	1,48	0,100	0,30
	AMP N2-P3a	−5,31	1,84	0,100	0,60

Fz	LAT N1	103,30	14,74	0,200	0,50
	AMP N1	−3,92	2,07	0,100	0,50
	LAT P2	155,50	15,01	0,200	0,60
	AMP P2	0,09	1,19	0,200	0,60
	LAT N2	199,52	16,98	0,100	0,30
	AMP N2	−2,93	1,08	0,090	0,40
	LAT P3a	294,64	31,48	0,100	0,40
	AMP P3a	2,04	1,25	0,300	0,30
	AMP N2-P3a	−4,96	1,73	0,030	0,30

RE: right ear; LAT: latency; AMP: amplitude; SD: standard deviation; ^*^ *P* < 0.05; Shapiro-Wilk Test, Person Test, and Spearman Test.

**Table 3:** Correlation between latency (ms) and amplitude (*μ*V), ERPs and HR in frequency protocol on the left ear.

LE	Variables	Mean	SD	*r*	*P*
Cz	LAT N1	97,55	13,65	0,080	0,40
	AMP N1	−4,70	1,84	0,090	0,20
	LAT P2	175,43	26,69	0,486	0,02^*^
	AMP P2	2,90	2,09	0,618	0,00^*^
	LAT N2	227,38	35,55	0,437	0,04^*^
	AMP N2	−1,32	1,18	0,100	0,60
	LAT P3a	282,06	32,19	0,090	0,90
	AMP P3a	1,92	2,17	−0,443	0,04^*^
	AMP N2-P3a	−3,29	2,72	0,200	0,60

Fz	LAT N1	99,04	15,66	0,100	0,50
	AMP N1	−4,58	2,09	0,090	0,80
	LAT P2	171,91	29,44	0,100	0,90
	AMP P2	1,17	1,92	0,516	0,01^*^
	LAT N2	214,24	35,06	0,523	0,01^*^
	AMP N2	−1,40	1,53	0,511	0,01^*^
	LAT P3a	277,70	25,54	0,100	0,70
	AMP P3a	2,11	1,59	0,200	0,90
	AMP N2-P3a	−3,51	2.10	0,090	0,50

LE: left ear; LAT: latency; AMP: amplitude; SD: standard deviation; ^*^ *P* < 0.05; Shapiro-Wilk Test; Person Test, and Spearman Test.

**Table 4:** Correlation between latency (ms) and amplitude (*μ*V), ERPs and HR in duration protocol on the left ear.

LE	Variables	Mean	SD	*r*	*P*
Cz	LAT N1	95,17	13,88	0,080	0,50
	AMP N1	−3,37	1,11	0,100	0,70
	LAT P2	151,19	12,42	0,100	0,90
	AMP P2	1,91	1,47	0,200	0,80
	LAT N2	207,30	18,52	0,090	0,50
	AMP N2	−2,54	1,54	0,050	0,70
	LAT P3a	283,74	23,15	0,200	0,70
	AMP P3a	2,34	1,97	0,100	0,90
	AMP N2-P3a	−4,88	2,25	0,090	0,50

Fz	LAT N1	93,34	22,10	0,060	0,40
	AMP N1	−2,79	1,69	0,200	0,80
	LAT P2	145,88	17,28	0,090	0,60
	AMP P2	0,81	1,39	0,090	0,80
	LAT N2	199,02	17,15	0,200	0,60
	AMP N2	−2,73	1,63	0,100	0,90
	LAT P3a	291,73	23,95	0,100	0,80
	AMP P3a	2,50	1,33	0,090	0,70
	AMP N2-P3a	−5,26	2,01	0,040	0,90

LE: left ear; LAT: latency; AMP: amplitude; SD: standard deviation; ^*^ *P* < 0.05; Shapiro-Wilk Test; Person Test, and Spearman Test.
